# Ecological indicators of mammal exposure to Ebolavirus

**DOI:** 10.1098/rstb.2018.0337

**Published:** 2019-08-12

**Authors:** John Paul Schmidt, Sean Maher, John M. Drake, Tao Huang, Maxwell J. Farrell, Barbara A. Han

**Affiliations:** 1 Odum School of Ecology and Center for the Ecology of Infectious Diseases, University of Georgia, Athens, GA 30602, USA; 2 Department of Biology, Missouri State University, 901 S. National Ave, Springfield, MO 65897, USA; 3 Cary Institute of Ecosystem Studies, 2801 Sharon Turnpike, Millbrook, NY 12545, USA

**Keywords:** boosted regression trees, comparative analysis, host, Ebola, frugivory

## Abstract

Much of the basic ecology of Ebolavirus remains unresolved despite accumulating disease outbreaks, viral strains and evidence of animal hosts. Because human Ebolavirus epidemics have been linked to contact with wild mammals other than bats, traits shared by species that have been infected by Ebolavirus and their phylogenetic distribution could suggest ecological mechanisms contributing to human Ebolavirus spillovers. We compiled data on Ebolavirus exposure in mammals and corresponding data on life-history traits, movement, and diet, and used boosted regression trees (BRT) to identify predictors of exposure and infection for 119 species (hereafter hosts). Mapping the phylogenetic distribution of presumptive Ebolavirus hosts reveals that they are scattered across several distinct mammal clades, but concentrated among Old World fruit bats, primates and artiodactyls. While sampling effort was the most important predictor, explaining nearly as much of the variation among hosts as traits, BRT models distinguished hosts from all other species with greater than 97% accuracy, and revealed probable Ebolavirus hosts as large-bodied, frugivorous, and with slow life histories. Provisionally, results suggest that some insectivorous bat genera, Old World monkeys and forest antelopes should receive priority in Ebolavirus survey efforts.

This article is part of the theme issue ‘Dynamic and integrative approaches to understanding pathogen spillover’.

## 1. Introduction

Since the first human case was identified in 1976, Ebolavirus has caused recurring human and animal outbreaks in Central Africa, and a major human epidemic in West Africa. Over the past two decades, the frequency of Ebolavirus spillover from animals to humans has increased [1] and caused steep reductions in wild populations of chimpanzees and gorillas in central Africa [2]. Despite accumulating outbreaks, animal hosts and viral strains (e.g. [3,4]), the basic ecology of Ebolavirus remains poorly understood [5]. In particular, reservoir species and the wider community of possible host species remain cryptic [2]. Though recent work suggests that spillover events (in mammal hosts, including human spillovers) appear to have seasonal triggers [6,7], the mechanisms driving spillover are still largely speculative.

Bats have long been viewed as reservoirs of Ebolavirus and other filoviruses [4,8–11]. Despite long-term asymptomatic infection of bats by Ebolavirus, and viral replication induced through experimental inoculation [12], live virus has not been found in any wild bat species to date [13]. In addition to bats, hosts from multiple other taxa may play a part in the maintenance and circulation of Ebolavirus [5]. Ebolavirus persistence and spillover may be influenced by variation in host community composition, depending on whether they contain species that serve as endemic hosts, resistant hosts or hosts supporting stuttering chains of transmission [13,14]. Bats may also be critical in transmitting the virus between hosts and over distances [2,3].

Human Ebolavirus transmission often arises from contact with infected wild mammals, which may thus serve to transmit infection [15]. Hosts may include species that are tolerant of long-term infections as well as those exhibiting high mortality rates from Ebolavirus infection. Given that previous human epidemics have been attributed to handling infected carcasses [16] and to contact with bats [9,17], identifying shared traits and spatio-temporal patterns among sylvatic hosts may offer insights about the ecological mechanisms that drive spillover infection in humans. Research on possible phylogenetic patterns in Ebolavirus competency, and the degree to which hosts comprise a group of species whose feeding niche or other habitat requirements tend to bring them into contact with Ebolavirus reservoirs, has significant potential to advance our understanding of the ecology and epidemiology of the virus in the sylvatic setting.

To identify characteristics that may confer a propensity for carrying Ebolavirus between unknown reservoirs and human populations, we compiled a list of mammal species tested for exposure to Ebolavirus and corresponding data on life-history traits, movement and diet. Using an automated approach to measuring variable importance, we predicted host status among 119 species and mapped the taxonomic distribution of presumptive Ebolavirus hosts in continental Africa.

## 2. Methods

### (a) Host status determination

We compiled a list of species representing either wild, captive, or domestic animals in Africa (*n* = 119) that have been tested for exposure to Ebolaviruses. We assigned a binary code to each of these mammal species according to their status as a host species known to be permissive to infection by any Ebolavirus as determined by antibody, RNA or viral assay—a subset of 23 species. In this way, host status was determined after an extensive literature search using key terms: *Ebola*, Host OR Reservoir OR Animal in Web of Science and EBSCO HOST through 2019. Results from the two literature repositories were combined and abstracts were read to determine whether animals were surveyed for Ebolaviruses, and if so, which Ebolavirus. If data were available in the abstract, we recorded location, species and Ebolavirus (or Ebolavirus strain) directly. If the abstract did not provide sufficient information, the full manuscript was examined for relevant details. We also compiled information from appendices on the methods by which hosts were tested for evidence of pathogen exposure and sample sizes. We restricted the final dataset to include only species on which laboratory assays were performed, excluding unverified evidence of Ebolavirus infection from animal mortality reportedly associated with human Ebola outbreaks or wildlife disease events, and to taxa identified to species.

### (b) Covariate traits

We used trait data from PanTHERIA [18] and from EltonTraits 1.0 [19] as covariates of host status. For all extant or recently extinct species with the class Mammalia, Jones *et al*. gathered 25 types of ecological and life-history information from the literature to create the 30 specific variables and 19 derived variables that make up the PanTHERIA dataset. After dropping traits with greater than 90% correlation with body mass (e.g. adult head body length and neonate body mass) and those covering fewer than 20 species, we included 29 variables related to life history, diet, activity and home range, which vary in their completeness across mammal species (table 1). EltonTraits provides literature-derived data on diet type/diversity, foraging strata, foraging time, and body size for extant bird and mammal species. Because EltonTraits interpolates data where literature values are unavailable, we restricted our use of EltonTraits to data on diet composition, which was unavailable from PanTHERIA. Continuous variables for life-history traits that spanned several orders of magnitude were log_10_-transformed.

**Table 1.** Trait covariates included in the final boosted regression tree model of Ebolavirus host status as a function of traits, weighted by study effort. Relative importance of each covariate was calculated by permutation tests.

covariate	relative importance
individuals sampled for viral RNA	21.4
latitudinal centre of range (degrees)	18.1
litter size	11.1
individuals sampled in sero-surveys	10.7
adult body mass (g)	7.4
gestation length (days)	6.5
fruit (% of diet)	5.4
diet breadth	5.4
social group size	5.0
weaning age (days)	4.6
habitat breadth	4.3

### (c) Sampling bias

To account for sampling bias, we tallied, based on sample sizes reported in primary studies, the number of individuals of each mammal species tested by each assay type: antibody, RNA (PCR) or live virus (electronic supplementary material). To control for the geographical likelihood of exposure, we included the latitude of the centroid of the range of each species using polygons from the International Union for the Conservation of Nature's terrestrial mammal range shapefiles (https://www.iucnredlist.org/resources/spatial-data-download).

### (d) Statistical analyses

#### (i) Boosted regression trees

We used boosted regression trees (BRTs) [20,21] with Bernoulli-distributed error for binary responses (Ebolavirus exposure, as indicated by any diagnostic method reported in primary literature). Boosted regression trees are a technique for learning the mapping between high-dimensional inputs and a unique response variable that has proved an effective approach to identifying functional trait associations in multi-host multi-pathogen systems [22]. The learning process consists of iterating regression trees, each defined by a series of recursive binary splits on randomly sampled predictor variables of mixed data types (e.g. categorical, numerical, binary). As this process is repeated, resulting trees are combined to create an ensemble model. We built 50 000 trees for each analysis reported here and present the most important variables for predicting Ebolavirus hosts. Because the dataset was relatively small (*n* = 119 species tested), we did not partition the data into training and holdout test sets. Instead, we applied fivefold cross-validation during model building to prevent overfitting and used permutation procedures to generate relative importance scores for each predictor variable. In these analyses, species that have not tested positive for Ebolavirus infection (*n* = 96) were designated non-hosts. This represents a conservative approach that minimizes type II error in a system where host status remains unknown without extensive field sampling. Analyses were performed using the *gbm* package [23] in R [24]. To gauge the effect of sampling bias on trait patterns, we also included sampling effort as covariates in the BRT model to predict host status by species. Finally, we restricted the BRT model to the top 10 most important covariates.

#### (ii) Phylogenetic signal

To assess the strength phylogenetic patterning in infection status for Ebolaviruses, we estimated phylogenetic signal as a measure of the statistical dependence among species' trait values owing to their phylogenetic relationships [25]. As infection status is a binary variable, we calculated the *D* statistic of Fritz & Purvis [26] using the function *phylo.d* in the ‘caper’ package in R [27] and the mammal supertree of Fritz *et al*. [28]. A *D* statistic equal to 1 indicates that the binary trait has a phylogenetically random distribution across the tips of the phylogeny, while a value of 0 indicates clumping expected by evolution under a Brownian motion model; however the values of *D* may fall outside of this range. To test for significant departure from each of these null models, a randomization test with 1000 permutations of the data is performed and compared to the estimated *D* statistic. Further, to assess phylogenetic signal in sampling intensity and predicted host status (both continuous variables), we used *fitContinuous* in the ‘geiger’ package in R to estimate Pagel's *λ* [29], a measure of phylogenetic signal that varies between 0 (phylogenetic independence) and 1 (phylogenetic dependence following pure Brownian motion along the observed tree).

## 3. Results

**Figure 1.**
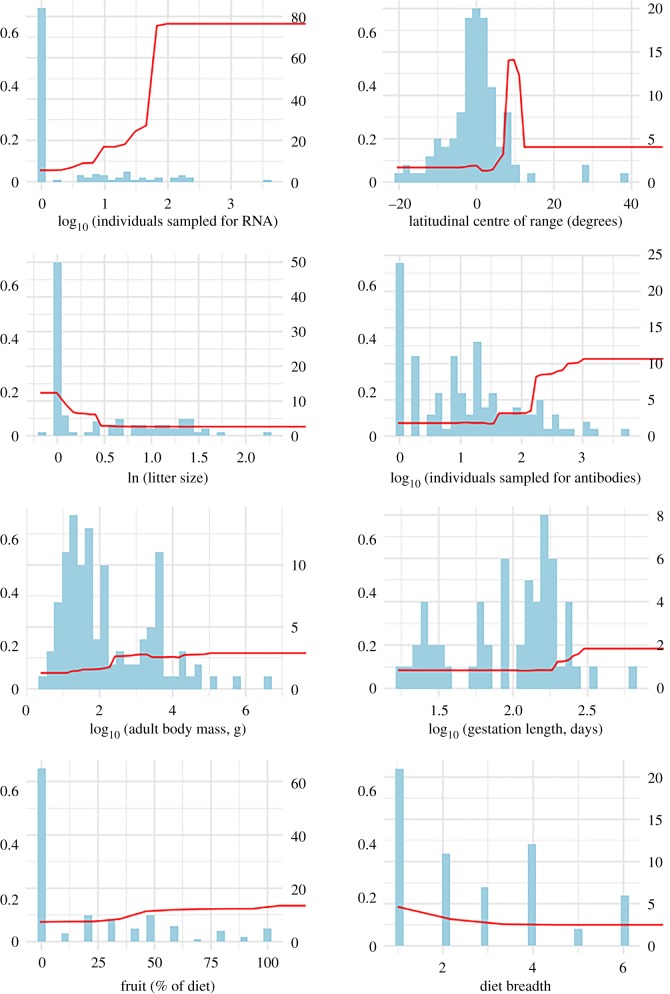
Partial dependence (red lines) of Ebolavirus host status as a function of the most important covariates in the final logistic BRT models overlaid on histograms of covariate distributions. *Left y*-axis represents output probabilities with the range indicating the magnitude of the effect, *right y*-axis represents counts of the number of species. (Online version in colour.)

**Figure 2.**
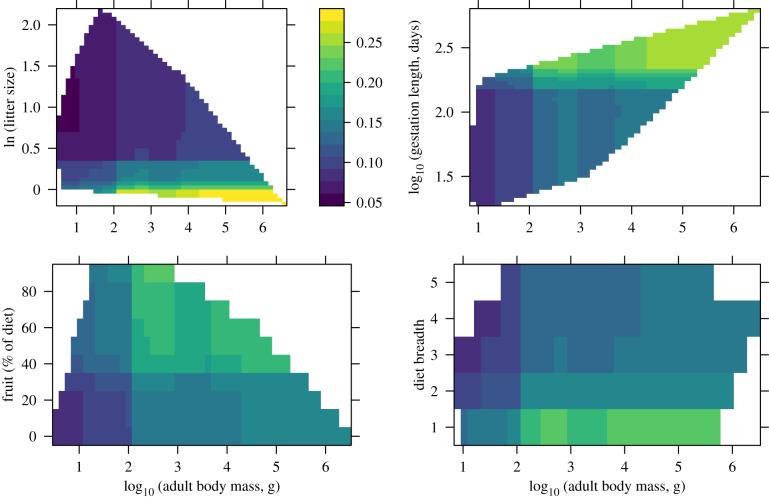
Joint partial dependence showing the interactive effects of predictors on Ebola host status in the final logistic BRT model. Each plot reflects a convex hull that constrains the prediction space to the range of values within observed covariate pairs. The legend shows increments of model output probability for all panels.

We were able to predict hosts with high cross-validation accuracy (AUC = 0.97) as a function of both species-level traits, centre of latitudinal range and sampling effort. Number of individuals sampled for Ebolavirus RNA was the most important predictor and strongly positively related to host status, as was the number of individuals sampled in sero-surveys, the fourth most important predictor (table 1). Hosts were most likely among species with ranges centred latitudinally between 4° and 7° N latitude, and were associated with small litters, large adult body sizes, long gestations, frugivorous diets, narrow diet breadth, early weaning, solitary or living in small social groups and narrow habitat breath (figure 1). To clarify the relationship between body mass and traits, we plotted the interactive effects of adult body mass on other relatively important life-history traits and diet (figure 2). The strongest interaction was between litter size and adult body mass such that hosts were most likely to be large-bodied with small litters and unlikely to be small-bodied with large litters. Although not as strong, gestation length and body mass interacted such that hosts had relatively long gestations and large body masses and, thus, were most likely among a small subset of species. Similarly, relatively large-bodied (more than 3 kg) frugivorous species were much more likely to be hosts than species with less than 40% frugivorous diet and lower body masses. The effect of diet breadth was also larger at adult body masses more than 3 kg. Trait patterns overall point to a slow pace of life and a frugivorous diet, but not necessarily to fruit bats. With the exception of duikers (*Philantomba monticola*, *Sylvicapra grimmia*, *Tragelaphus scriptus*), several rodents (*Cricetomys emini*, *Anomalurus derbianus*) and the elephant (*Loxodonta africana*), species with litter sizes of approximately 1 are nearly all bats. Of these, only *A. derbianus* was Ebolavirus-positive. While the majority of Ebolavirus-positive species (table 2) were either bats or primates, and both families include many frugivores, highly frugivorous (greater than 40% of diet) species are also found among the rodents, ungulates, and carnivores.

**Table 2.** Total number of species surveyed, total number of species testing positive for Ebolavirus exposure, and total number of individuals sampled by mammal order.

mammal order	total species surveyed	total positive species	total individuals sampled
Artiodactyla	6	1	67
Carnivora	6	0	36
Chiroptera	43	14	13 016
Hyracoidea	1	0	14
Macroscelidea	2	0	57
Primates	15	5	678
Proboscidea	1	0	2
Rodentia	34	2	4682
Eulipotyphla	11	1	231

The phylogenetic signal in host status, as measured by the *D* statistic, was estimated to be 0.53 and indicated significant phylogenetic clumping intermediate between phylogenetic randomness (*p* = 0.001) and pure Brownian motion (*p* = 0.04). Phylogenetic signal in sampling intensity was found to be fairly low (*λ* = 0.134), while phylogenetic signal in the predictions from the BRT was found to be relatively high (*λ* = 0.447). Overall, in our dataset relatively few non-bat species have tested positive (table 2), and none as a result of virus isolation. This is likely owing to the variation existing among bats and across mammal clades with known Ebolavirus hosts. Of 76 non-bat mammal species assayed to date in our dataset, 10 have tested positive (total sampling effort ≅5536) for Ebolavirus infection, as compared to 26 of 99 bat species (total sampling effort ≅ 13 016). Clearly, the disproportionate representation of bats among known Ebolavirus hosts is, in part, owing to disproportionate sampling of bats compared to other animals, and across the mammal tree, variability in the prevalence of hosts among species surveyed must reflect to some degree phylogenetic patterns in sampling biases. Yet, large sampling biases notwithstanding, we find a role for (i) life-history features such as gestation length that may be linked to immune function that, in turn, determine survivorship, rates of viral shedding, and transmission; (ii) ecological factors related to exposure, such as fruit consumption; and, (iii) interactions or interrelationships between diet or other ecological factors and life-history.

## 4. Discussion

### (a) Taxonomic patterns and sampling bias

Ebolavirus hosts are scattered across a number of distinct mammal clades, but concentrated among Old World fruit bats, primates and artiodactyls. Critically, these patterns must be considered in light of very biased sampling effort across mammal species. Taxonomic subgroups differ greatly not only in the number of species reported to have been sampled in the published literature, but also in the number of individuals sampled per species. Olson *et al*. [30] found that bats represented 61% and rodents 27% of the 8040 mammals tested in sero-surveys, and 30% and 48% of 5309 mammals surveyed for viral RNA from 1976 to 2011. Detection of Ebolavirus sequences in members of Pteropodidae in 2005 skewed study effort toward these prime suspects. Reported sampling of just six species (*Eidolon helvum*, *Epomophorus gambianus*, *Rousettus aegyptiacus*, *Micropteropus pusillus*, *Epomops franqueti*, *Hypsignathus monstrosus*) exceeds 1500 individuals, yet fewer than 1000 individuals had been sampled in 20 other (insectivorous) bat species [2]. Publication bias against negative results may offset these apparent sampling imbalances to the extent that non-host species within our dataset have actually received greater sampling effort than is reflected in the literature. Nonetheless, documentation of those species surveyed, but found to be Ebolavirus-negative, is likely to be very incomplete, exacerbating biases in the record of which species and taxonomic groups have been investigated [13].

Although bats have been strongly suspected, evidence that human outbreaks have resulted from contact with bats is, so far, indirect. As the only set of species that have shown replication and high circulating titres of Ebolavirus without accompanying illness [12], bats appear to be capable of functioning as reservoirs. Moreover, detection of virus in lung tissues and faeces suggests that bats could transmit virus to other susceptible species via multiple routes [1,3]. On the other side, recent experimental inoculations of Egyptian fruit bats (*Rousettus aegyptiacus*) with Ebolavirus strains [31,32] resulted in low levels of detectable viral RNA suggesting that fruit bats could, instead, be dead end hosts incapable of transmitting. Circumstantially, bat consumption associated with an annual bat migration has been tied to the index case of an Ebolavirus disease outbreak (May 2007) in the Democratic Republic of Congo [16]. As further circumstantial evidence, an insectivorous bat (*Mops condylurus*) tested positive for Ebolavirus-specific antibodies [33], survived experimental infection while showing high viremia [12] and, although evidence is anecdotal rather than serological, *M. condylurus* may have been the source of infection to the human index case in the 2014 West African Ebola epidemic [13,17]. However, with little market as bushmeat [34], direct human contact with insectivorous bats may be more limited than with fruit bats or rodents. And, despite the importance of fruit bats in bushmeat markets across Africa [34], no fruit bat hunter has yet been identified as the index case in any Ebolavirus disease outbreak [2,34,35], nor has any rodent species been directly tied to Ebolavirus spillover despite widespread exploitation of rodents for food [36]. Thus, although thousands of individuals have been tested, live virus has never been isolated from wild bats or rodents [13].

Survey type presents an additional issue in weighing the evidence of exposure to Ebolavirus across the mammal tree. In a review of studies, only bats, rodents and carnivores had tested positive in sero-surveys [30]. Few positives were detected in the first two groups (3.2%, 0.04%), whereas carnivores yielded a 24.1% positive rate despite fewer animals being sampled—largely a function of domestic dogs. In RNA surveys, only bats have tested positive, but at a low rate overall (0.9%). By far the strongest evidence of exposure and potential to serve as hosts comes from surveys of live virus from mammal carcasses. Of 33 non-human primates, 13 artiodactyls, six carnivores and two proboscideans surveyed, over half the primates and 7.7% of artiodactyls tested positive. Furthermore, strong evidence directly links Ebolavirus-infected primate and duiker carcasses to the initiation of human outbreaks [16]. Great apes and duikers are, therefore, the only firmly established Ebolavirus hosts.

### (b) Potential role of frugivory

Ebolavirus transmission to wildlife via fruit contamination by infected bat saliva or faeces has been a prevailing hypothesis [8]. But frugivory could also be associated with Ebolavirus transmission through a variety of mechanisms. Fruiting phenology, often corresponding to dry seasons when other food resources may be scarce, could increase interspecific contact and probability of transmission. In addition to support for seasonal shifts from wet to dry conditions as triggers of Ebolavirus spillover [6,7], Ebola outbreaks in apes are known to have occurred in dry seasons, during which fruit consumption made lead to direct or indirect contact between primates and bats [8,37,38]. Recent analyses of viral sequences suggest that primates are more likely to be reservoirs of the Tai Forest and Bundibugyo Ebolaviruses than bats [39]. This, combined with recent observations of gorilla foraging (successive temporal foraging on fruiting trees) suggests that Ebolavirus transmission is linked to seasonally dynamic behaviours in primates that centre on resource availability [40]. Reliance on seasonally available resources may also affect the timing of pregnancy in primates [41,42], which may have implications for susceptibility. Finally, over 70% of animals harvested for bushmeat in the moist forests of West and Central Africa were frugivorous to some degree, suggesting that bushmeat of frugivorous mammals is more likely to present a transmission pathway of Ebolavirus to humans [36].

### (c) Role of host body size and life history

Hosts that tested positive for Ebolavirus tended strongly toward large bodies. This may be attributable to several mechanisms. Larger hosts: (i) have potentially higher contact rates with humans via bushmeat hunting and consumption—although more rodents less than 15 kg were harvested, mammals more than 15 kg comprised more than half of bushmeat biomass in a survey of tropical Africa by Fa *et al*. [36]; (ii) can range further, leading to potentially greater contact with disease agents (e.g. infected carcasses, contaminated fruit, or directly with other infected hosts)—although compared to other variables related to body size and diet, home range size was relatively unimportant as a predictor in the BRT model; (iii) are more conspicuous and may shed or release greater quantities of virus, leading potentially to an increased likelihood of being encountered by humans or other mammals when infected or dead; and (iv) have slower life histories, the immune correlates of which may further increase the infectious period and the potential for transmission to other species, including humans. While all four factors may play some part, the relationship of body size to the quantity of virus generated, the detectability of dead or moribund hosts in the environment and the immune correlates of size may be particularly important to susceptibility and transmission.

The importance of gestation length and small litters in the BRT model suggest that slow pace of life, more typical of large mammals, may influence susceptibility. Pregnancy in bats, typically small-bodied but with slow life histories, has been linked to both high seroprevalence and seasonal spillover of Hendra, Nipah and Ebola viruses [37], suggesting that gestation may be linked to increased risk of viral shedding. Importantly, in BRT analyses, Ebola hosts were more likely among species with gestation lengths more than 100 days across the range of adult body masses (figure 2). Thus, gestation length itself, rather than simply standing in for life history broadly, may be directly related to susceptibility.

### (d) The use of boosted regression trees for comparative analyses

**Figure 3.**
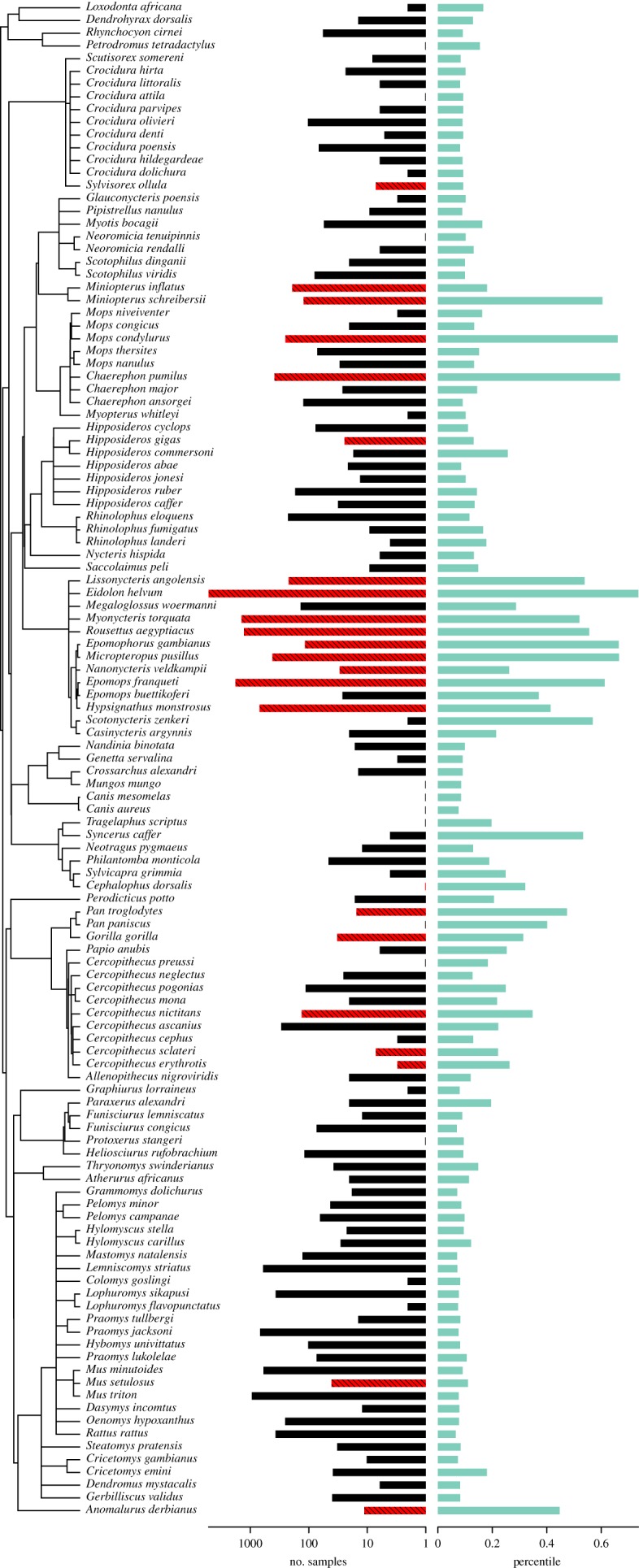
Trees showing the phylogenetic clustering of species exposed to Ebolavirus (*n* = 119). *Left* bars indicate sampling effort (*black-hatched red* indicates positive), and *right* bars percentile rank of predicted risk of exposure/infection. (Online version in colour.)

Statistical inference in comparative studies is generally complicated by phylogenetic non-independence among species [43]. In regression analyses of comparative data, such as phylogenetic generalized least squares, the current best practice is to estimate the degree of phylogenetic non-independence jointly with the regression coefficients, allowing for us to optimize the error structure of the residuals [44]. However, current BRT approaches cannot do simultaneous estimation of phylogenetic non-independence. While we show that BRTs can have high predictive accuracy for comparative data, the lack of phylogenetic comparative methods for BRTs means that we should be cautious when making strong statistical statements regarding relationships among traits. However, the results of BRTs for comparative data are still beneficial to report, and the phylogenetic signal in predicted host status indicates that influential traits revealed by the BRT suggest there are lineages with high potential for being Ebolavirus hosts. Our results thus warrant the development of phylogenetic comparative methods for GBMs to allow more robust inferences of these important traits (figure 3).

### (e) Optimizing future survey efforts to identify wild hosts of Ebolavirus

Taxonomically, species with the highest predicted probabilities of serving as Ebolavirus hosts that have, so far, not tested positive for Ebolavirus exposure are mainly insectivorous bats (*Chaerephon*, *Hipposideros*, *Myotis*, *Pipistrellus*, *Rhinolophos*), cercopithid apes and forest antelopes (*Neotragus*, *Philantomba*, *Tragelaphus*), suggesting that these groups may deserve priority in future surveys. However, among species with the lowest predicted probabilities of serving as Ebolavirus hosts that have, so far, not tested positive for Ebolavirus exposure, the best sampled are mainly small- to medium-sized rodents (*Mus*, *Lemniscomys*, *Rattus*, *Lophuromys*, *Oenomys*, *Mastomys*, *Hybomys*, *Praomys*, *Pelomys*, *Gerbilliscus*, *Thryonomys*, *Steatomys*, *Hylomyscus*, *Paraxerus*) and insectivores (*Crocidura*), suggesting that these groups should be a low priority for future surveys.

## Supplementary Material

Supplementary data files

## Data accessibility

All data and code to reproduce these analyses are available in the electronic supplementary material.
